# Swimming exercise improves short‐ and long‐term memories: Time‐course changes

**DOI:** 10.14814/phy2.14851

**Published:** 2021-06-10

**Authors:** Mahmoud A. Alomari, Karem H. Alzoubi, Omar F. Khabour

**Affiliations:** ^1^ Department of Physical Education Qatar University Doha Qatar; ^2^ Division of Physical Therapy Department of Rehabilitation Sciences Jordan University of Science and Technology Irbid Jordan; ^3^ Department of Clinical Pharmacy Jordan University of Science and Technology Irbid Jordan; ^4^ Department of Medical Laboratory Sciences Jordan University of Science and Technology Irbid Jordan

**Keywords:** exercise, learning, maze, memory, time course

## Abstract

The beneficial effects of exercise training on memory formation are well documented. However, the memory enhancement profile following the time‐course of exercise training remains unknown. In this investigation, changes in the spatial hippocampal memory following a time‐course of swimming exercise training were examined. Young adult Wistar rats were tested for both short‐term and long‐term memories using the radial arm water maize (RAWM) paradigm following 0, 1, 7, 14, and 28 days of swimming exercise training (60 min per day, 5 days/week)s. The mean total errors on RAWM during the learning phase and memory testing remained the same (*p *> 0.5) after 1 day of swimming exercise. On the other hand, swimming exercise‐induced significant enhancement to the learning phase and memory formation after 7 days of training (*p *< 0.01). Errors decreased (*p *< 0.0001) after 7 days of training and remained lower (*p *< 0.0001) than baseline without differences between 7, 14, and 28 days (*p *> 0.5). Similarly, short‐ and long‐term memories improved after 7 days (*p* < 0.05) of training as compared to the baseline without differences between 7, 14, and 28 days (*p* > 0.05). The time course of improvement of learning and both short‐ and long‐term memories after swimming exercise were evident after 7 days and plateaued thereafter. Results of the current study could form the base for future utilization of exercises to enhance cognitive function in healthy individuals.

## Graphical abstract

Memory enhancements profile following the time‐course of exercise training remains unknown. Both short‐term and long‐term memories were examined following 0, 1, 7, 14, 28 days of exercise swimming training. The current findings show the time course of improvement of short‐ and long‐ term memories after swimming exercise. Interestingly, these improvements were evident after 7 days of exercise and plateaued thereafter.

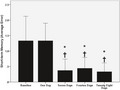

## 1 INTRODUCTION

The benefits of exercise training are numerous and well documented. It protects against serious illnesses including cardiovascular diseases, diabetes, cancer, and neurodegenerative diseases (Ji et al., 2021; Quindry & Franklin, 2021; Nagpal & Mottola, 2020). Exercise can also improve body immunity, neuromuscular performance, sleeping time, and individual mood (Kelley & Kelley, 2017; Mikkelsen et al., 2017; Simpson et al., 2020).

Ample evidence showing the importance of exercise for cognitive function has accumulated (Hotting & Roder, 2013; Intzandt et al., 2018; Mikkelsen et al., 2017). For example, exercise protects animals from the decline in cognition associated with aging (Kim et al., 2019). In addition, voluntary and forced exercises were shown to enhance memory formation (Alomari et al., 2016). The cognitive benefits of exercise have been demonstrated across all ages (O'Leary et al., 2019) and attributed to changes in gene expression (Kim et al., 2019). Furthermore, a recent study on mice showed that exercise benefits could be transmitted from parents into progeny (McGreevy et al., 2019). Similar findings were observed in humans. Exercise protects individuals from cognitive decline associated with aging and diseases (Lopez‐Fontana et al., 2018). In addition, exercise can improve cognitive function in adolescents, adults, and the elderly (Fernandes et al., 2018; Marston et al., 2019; McSween et al., 2019; Stern et al., 2019).

Acute and chronic exercise training are both associated with improved cognitive function (Loprinzi et al., 2018; McSween et al., 2019; Suarez‐Manzano et al., 2018; Wheeler et al., 2019). In a meta‐analysis involving 25 studies, exercise occurring before memory encoding and during early memory consolidation enhanced episodic memory function (Loprinzi et al., 2019). However, changes in the magnitude of cognitive function following a time‐course of exercise training are yet to be investigated. Therefore, the current investigation aimed to examine spatial hippocampal memory following a time‐course of exercise training using the radial arm water maize (RAWM) paradigm.

## 2 METHODOLOGY

### 2.1 Animals and design

Male Wistar rats (weight: 180–220 g) were used in the study. The rats were kept in stainless steel wired cages for 2 weeks prior to the exercise intervention for acclimating to a 12:12 light/dark cycle at 24±1°C temperature. Sanitized water and regular rodent food were supplied to the rats while in the cages throughout the study period. Caring, feeding, exercise, and memory testing were conducted in the animal care facility of Jordan University of Science and Technology, Irbid, Jordan.

Five groups of 12 animals each were used in the experiments. The rats were administered a swimming exercise program 60 min per day, 5 days/week. Each group was exercised for either 0, 1, 7, 14, or 28 days. The RAWM paradigm (Alzoubi et al., 2019; Alzoubi et al., 2019; El‐Elimat et al., 2019) was used to determine spatial memory for each group at its corresponding time point (0, 1, 7, 14, or 28 days). Memory was determined after 30 min (short‐term memory) and 5 h (long‐term) of finishing the exercise session (Alqudah et al., 2018). The experimental procedures were reviewed and approved by the Institutional Animal Care and Use Committee of Jordan University of Science and Technology.

### 2.2 The exercise training protocol

The rats were subjected to a swimming exercise protocol in a cylinder tank. During the exercise program, the rats were alternated between 5 min of resting and swimming for 60 min. After swimming, each rat was removed immediately from the tank, dried with a piece of cloth, and placed for resting in a cage. The tank was 50 × 35 × 35 cm in height, diameter, and water depth, respectively (Khabour et al., 2013).

### 2.3 Memory testing

The RAWM paradigm was used for evaluating spatial memory in a dimly lit room (Alzoubi, Mayyas, et al., 2019; Alzoubi et al., 2019; El‐Elimat et al., 2019). The paradigm is a black circular vessel that contains preserved water at 24 ± 1°C. Inside the vessel, six‐V‐shaped stainless steel sheets forming a central area connected to six arms for swimming, one of which is designated as the goal arm. A platform is hidden at 2 cm underwater at the far end of the goal arm (Alquraan et al., 2019).

The rats performed two sets of six consecutive attempts separated by 5 min of resting to assure learning the paradigm, including the goal arm location. Subsequently, short‐ and long‐term memories were examined at 30 min and 5 h, respectively. During the learning attempts, each animal was allowed to swim freely in the RAWM paradigm to find the hidden platform within 1 min. Once on the platform, the rat was given 15 s to observe visual cues before removing it off the platform to resume the next trial. Visual cues were placed in the same positions during the experiment. The rat was guided toward the platform to observe the cues for 15 s when was unable to find the platform within the permitted 1 min.

An error was scored when the rat arrived at the wrong arm during the 1‐min‐search for the platform. An entry to an arm was recorded, when the whole body of the rat (excluding the tail) is inside the arm. Memory tests were administered in a similar pattern as to the acquisition trials. However, in the memory tests, animals were neither guided to the goal arm, nor observed cues for 15‐s while on the hidden platform. Instead, once the rat reached the platform, it was returned to the home cage immediately. During testing for memory, all animals reached the hidden platform within <1 min.

### 2.4 Statistical analyses

Statistical analyses were completed with SPSS software for Windows (version 22.0). Data are expressed as mean ± SD, and α was preset at *p* < 0.05.

One‐way repeated measure ANOVA was used to compare the average number of errors committed by the rats to determine the change short‐ and long‐term memories during the time course of the study.

## 3 RESULTS

### 3.1 Learning effect

**FIGURE 1:**
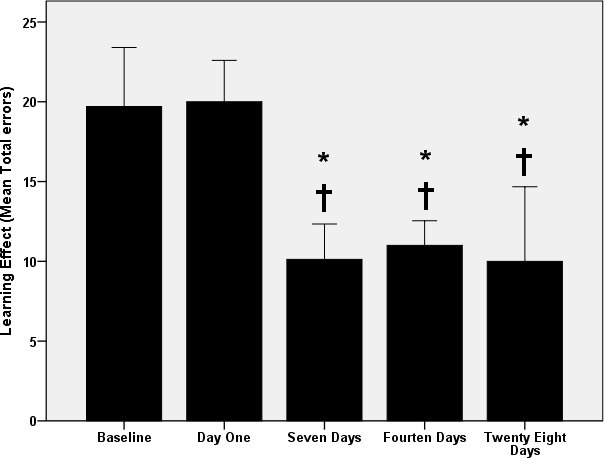
Changes in spatial learning with swimming exercise. *: *p* < 0.05 versus baseline. †: *p* < 0.05 versus 1 day. Data are presented as mean ± SD (*n* = 12 rats/group)

The ANOVA presented in Figure 1 shows differences (*p* < 0.001) in spatial learning among the rats. According to the post hoc analysis, the mean total errors on RAWM remained the same (*p* > 0.5) after 1 day of swimming exercise. However, errors decreased (*p* < 0.001) after 7 days and remained lower (*p* < 0.001) than baseline and 1 day of exercise without differences between 7, 14, and 28 days (*p* > 0.5).

### 3.2 Effect of swimming exercise on memory

**FIGURE 2:**
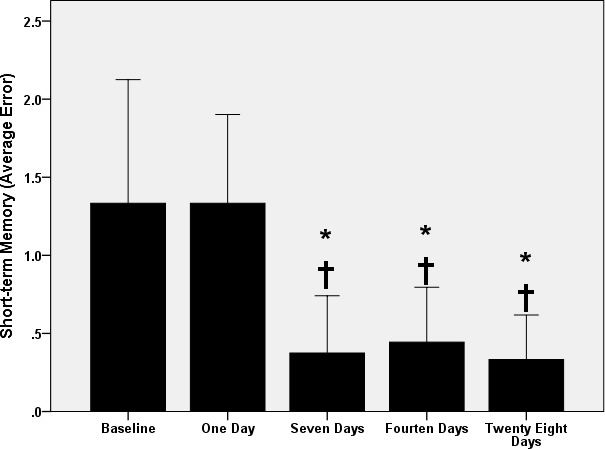
Short‐term memory changes with swimming exercise. *: *p* < 0.05 versus baseline. †: *p* < 0.05 versus 1 day. Data are presented as mean ± SD (*n* = 12 rats/group)

The one‐way ANOVA revealed changes in short‐ (*p* < 0.009) and long‐term (*p* < 0.004) memories with exercise. Post hoc analysis showed that the short‐term memory remained unchanged (*p* > 0.5) after 1 day of exercise (Figure 2). However, the number of errors committed, on the RAWM, decreased after the 7th (*p* < 0.02), 14th (*p* < 0.03), and 28th (*p* < 0.008) days of exercise as compared to the baseline without differences (*p* > 0.5) between 7th, 14th, and 28th days of the exercise time‐course.

**FIGURE 3:**
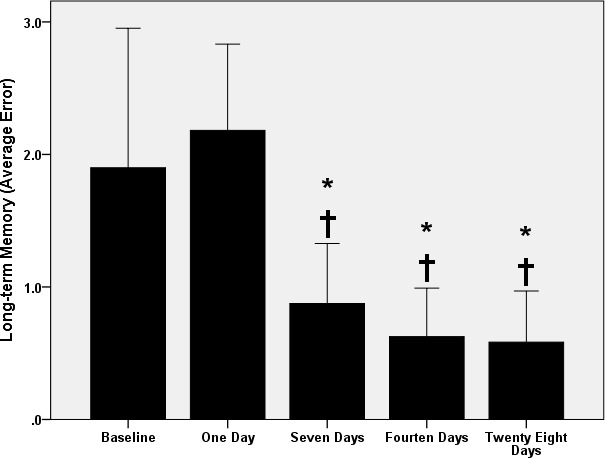
Long‐term memory changes with swimming exercise. *: *p* < 0.05 versus baseline. †: *p* < 0.05 versus 1 day. Data are presented as mean ± SD (*n* = 12 rats/group)

Another post hoc analysis showed that the long‐term memory remained unchanged (*p* > 1.0) after 1 day of exercise (Figure 3). However, the number of errors committed, in the RAWM, decreased after the 7th (*p* < 0.04), 14th (*p* < 0.01), and 28th (*p* < 0.004) days of exercise as compared to the baseline without differences (*p* > 0.05) between 7th, 14th, and 28th days of exercise (Figure 3).

## 4 Discussion

The study examined the time‐course effect of swimming exercise on memory in rats. The results revealed that swimming can improve short‐ and long‐term memories similarly. Improvements in short‐ and long‐term memories were evident after the 7th day of exercise then plateaued thereafter (i.e., after 14 and 28 days of exercise) without changes after one day of exercise. The study confirms the importance of exercise for memory. Additionally, the plateau indicates that the improvements in memory seem to be rapid. However, more studies are needed to determine changes in memory after voluntary and forced exercise in animals and humans with more time points and longer periods. Furthermore, studies are needed to investigate the sustainability of exercise training on memory with training cessation (i.e., detraining). Examining the mechanisms for these exercise‐induced adaptations in memory is also warranted.

The advantages of exercise are now undeniable. Cardiovascular, respiratory, metabolic, immunological, neuromusculoskeletal, mental, and social benefits can be gained from regular engagement in exercise (Lahart et al., 2019). Similarly, evidence of neurocognitive improvements of exercise has recently accumulated (Lee et al., 2019). Regular participation in exercise seems to enhance academic‐ and work‐related achievements among children, and young and old adults, with and without cognitive deficits. These achievements have been attributed to enhanced information perception, retention, and retrieval in verbal, mathematic, and memory tasks (Raichlen & Alexander, 2017). Interestingly, the benefits were related to the amount of time committed to physical activities (Raichlen & Alexander, 2017). These improvements are usually combined with neural structural and functional alterations. Among these alterations are enlarged brain parts (Sexton et al., 2016), enhanced brain connectivity (Li et al., 2017), and increased cerebral and hippocampal blood flow (Steventon et al., 2019), coupled with neurogenesis (Voss et al., 2019).

In animals, exercise also enhances data acquisition, retention, and retrieval (Mello et al., 2008). These cognitive adaptations are associated with cerebral neurogenesis, angiogenesis, and increased brain volume and activity, synaptic plasticity, cerebral blood flow, and spine density (Stimpson et al., 2018). The molecular bases of these adaptions have been attributed mainly to neutrophils, particularly BDNF (Kondo, 2017).

Uniquely, the current improvement was evident after 7 days of exercise without additional development of cognitive function after 14 and 28 days of exercise. These findings indicate that the improvements are rapid and can level‐off even if the exercise of the same components (i.e., type, intensity, frequency, and duration) persisted. The exercise intervention was performed 5Xs/week for 60 min throughout the study period. Maybe, this exercise program was sufficient enough for the first week of the exercise program; thus an increase in the exercise program intensity, frequency, and duration might be needed to induce further adaptations. Therefore, more studies with gradual progressive exercise programs are warranted.

The short‐term effect of exercise on cognitive function measures is sparse. In animals, a single bout of low, but not high, intensity exercise can improve memory during the early stage of brain injury in rats (Yoon & Kim, 2018). In healthy rats, however, memory improvements were evident after 2 weeks of exercise (Lovatel et al., 2012). In a study for Berchtold and colleagues, improvements in memory were shown after 3 weeks of daily exercise and persisted thereafter, however gradually decreased to baseline level, after 1–2 weeks of detraining (Berchtold et al., 2010). These improvements were observed in adult rats after 4, but not 2, weeks, while were observed in adolescent rats after 2 and 4 weeks of exercise (Hopkins et al., 2011). According to these studies, the time‐course adaptations in memory to exercise are still uncertain and seem to be exercise time‐, protocol‐, and age‐dependent. Therefore, more studies are needed to examine the contribution of exercise time and protocol as well as age on memory.

The precise mechanism for these cognitive adaptations is still elusive; a cascade of neural adjustments, however, has been proposed. Rhythmic muscular contraction during exercise seems to stimulate the growth of neurons in certain brain compartments (i.e., hippocampus and cerebral cortex) essential for learning and memory to release BDNF. Subsequently, BDNF seems to induce neural plasticity thus enhances cognitive function, learning and memory (Hopkins et al., 2011). However, these are mere speculations in need for substantiation in future mechanistic studies.

In conclusion, the current findings show the time‐course of improvement of short‐ and long‐term memories after swimming exercise. Interestingly, these improvements were evident after 7 days of exercise and plateaued thereafter. The results could form the base for future utilization of swimming exercises to ameliorate neuropsychiatric diseases‐induced learning and memory impairment.

## CONFLICT OF INTEREST

The authors declare no conflict of interest.

## AUTHORS CONTRIBUTIONS

MA conceived and designed the experiments; analyzed and interpreted the data; contributed reagents, and materials, and wrote of the paper. KA participated in designing the experiments; analyzing and interpreting the data; contributed reagents, and materials, and helped in writing the paper. He also performed the experiments. OK participated in designing the experiments; analyzing and interpreting the data; and helped in writing the paper.
